# The effect of mouthwashes on fluoride dentifrices in preventing dental abrasion or erosion

**DOI:** 10.25122/jml-2020-0112

**Published:** 2021

**Authors:** Rachna Raj, Safia Haideri, Bipin Kumar Yadav, Joohi Chandra, Reema Malik, Amit Raj

**Affiliations:** 1. Department of Public Health Dentistry, Patna Dental College and Hospital, Patna, Bihar, India; 2. Department of Pedodontics and Preventive Dentistry, Patna Dental College and Hospital, Bankipur, Patna, Bihar, India; 3. Department of Dentistry, Uttar Pradesh University of Medical Sciences, Saifai, Etawah, India; 4. Department of Periodontology Implantology, ITS Center for Dental Science and Research, Muradnagar, Gaziabad, Uttar Pradesh, India; 5. Department of Conservative Dentistry and Endodontics, Maulana Azad Institute of Dental Science, New Delhi, India; 6. Department of Prosthodontics, All India Institute of Medical Sciences, New Delhi, India

**Keywords:** abrasion, erosion, fluoride toothpaste, mouthwash

## Abstract

Erosive tooth wear (ETW) refers to the chemical dissolution of mineralized tissues by acids of non-bacterial origin. It occurs in the primary as well as the permanent dentition. In this study, our objectives were to investigate and compare the impact of chlorhexidine gluconate (CHX), essential oils (EO), and cetylpyridinium chloride (CPC) on ETW protection produced by conventional fluoride kinds of toothpaste. A clinically relevant in-vitro erosion/abrasion pH cycling model was employed to test the effect of the aforementioned mouthwashes on modulating the ability of NaF and SnF2 types of toothpaste. The mean dentin surface loss associated with NaF toothpaste was significantly lower than for the SnF2 toothpaste. On the other hand, enamel surface loss with SnF2 toothpaste was significantly lower than for the NaF toothpaste. Also, the surface loss of erosion was significantly higher when associated with abrasion than without brushing and for both enamel and dentin. There was no significant difference in the surface loss among all types of mouthwash. Commonly used types of mouthwash containing antimicrobial agents or additional fluoride do not impact fluoride toothpaste action on erosion/abrasion. Also, considering erosion only, the tested SnF2 dentifrice provided better protection against surface loss of enamel than the other.

## INTRODUCTION

Recent literature has depicted that the cumulative results of abrasion and erosion in humans surpass the single effect by approximately 50%. This can be due to the fact that weak enamel is more prone to be affected by physical forces [1]. The proper diagnosis, guidance, motivation by the assessing dentist, and compliance by the affected subjects to the prescribed treatment and precaution towards avoidance of further loss can aid in preventing further tooth loss by the wasting diseases. The removal of the causative factor proves to be the best method of preventing enamel wear [2]. Also, symptomatic and etiological treatment with preventive measures can minimize tooth wear [3].

Fluoride is considered one of the most effective measures in preventing adverse effects on enamel, and it should be researched for assessing its efficacy in preventing enamel erosion [4]. In previous studies, dentifrices with fluoride as a component have been proven effective in remineralization of incipient enamel erosions and provide resistance against future enamel erosion [5, 6]. Also, dentin wear caused by abrasion or erosion is shown to be markedly reduced with using dentifrices having sodium fluoride as a component in a 1–100 ppm concentration. However, this effect did not markedly increase in proportion to the fluoride concentration [7]. More effective and long-term results are shown by stannous fluoride-containing dentifrices in terms of erosion prevention [8]. The action of the stannous fluoride ion is shown by protective layer formation on enamel having calcium fluoride, calcium stannous fluoride, SnF3PO4 and Sn2(PO4)OH [9].

In the general population, tooth brushing using toothpaste having fluoride along with mouthwash rinsing is the most common oral hygiene routine. Using mouthwash with antimicrobial effects proved effective against dental caries, gingivitis, and periodontitis [10]. Various commercially available mouthwashes contain different formulations having essential oils, chlorhexidine, and/or cetylpyridinium chloride. Lower pH of various mouthwashes may aid in increasing enamel erosion. However, the mechanism behind their cumulative effect with fluoridated toothpaste is still unknown. It is hypothesized that using mouthwash after toothpaste may diminish the effect of anti-erosive agents by dissolving the fluoride attached to the teeth due to their low pH [11]. However, a simultaneously antibacterial agent in the mouthwashes may help in enhancing the effect of fluoride in the enamel, owing to their high affinity for teeth structures.

Hence, the present study was an attempt to employ a confirmed model for tooth wear (abrasion and erosion) to evaluate the efficacy of mouthwashes containing essential oils, cetylpyridinium chloride and chlorhexidine in the protection against enamel tooth wear corresponding to two kinds of toothpaste having different fluoride composition.

## MATERIAL AND METHODS

This was an experimental comparative trial performed at the Government Dental College, Patna, Bihar, India. In this study, the design included the usage of two types of fluoridated toothpaste and four rinses in tests and controls, as well as tooth brushing and no tooth brushing in erosive conditions. The study design was evaluated and assessed for both dentin and enamel separately. In this study, mouth rinses with essential oils, cetylpyridinium chloride, and chlorhexidine, rinsing with deionized water or fluoride, and fluoridated toothpaste with either sodium fluoride or strontium fluoride were tested.

Study samples were collected from the dentin and enamel of bovine animals. The collected samples were then put through the pH cycling model for 5 days, where the samples were treated with a toothpaste having either sodium fluoride or strontium fluoride two times a day, followed by subjection to different mouth rinses with 5 cycles of erosion per day. To simulate the experiment in natural conditions, remineralization was done in an artificial salivation environment. After completing the five-day cycle, loss on the dentinal and enamel surfaces was evaluated with profilometry (non-contact) along with an assessment of the effectiveness of the different treatment protocols employed.

The present study also evaluated the prevention in the tooth wear shown by the sodium fluoride and stannous fluoride toothpaste in addition to different mouth rinses. In this study, 5 different types of mouthwash were employed, which were selected on the basis of their easy availability, patient preference, and dentist endorsement. For two fluoridated kinds of toothpaste, Senquel-F was chosen to have sodium fluoride and Crest with stannous fluoride, owing to their popularity and availability. The five different types of mouthwash were:

- CHX: Chlohex ADS® Antiseptic and Antiplaque Mouthwash. Active Ingredients: Chlorhexidine Gluconate 0.2 % (Group Pharmaceuticals Ltd, India);
- EO: Listerine® Original Mouthwash. Contents: Eucalyptol 0.092%, Menthol 0.04%, Methyl salicylate 0.060% and Thymol 0.064% (Johnson & Johnson, India);
- CPC: Colgate® Plax Complete Care Mouthwash. Contents: Cetylpyridinium Chloride 0.075%, Sodium Fluoride 0.05% (Colgate-Palmolive, India);
- F: Listerine® Cavity Fighter Mouthwash. Active Ingredients: Eucalyptol 0.092%, Menthol 0.04%, Methyl salicylate 0.060%, Thymol 0.064% and NaF (Johnson & Johnson, India);
- Distilled water as a control (negative).

To be used as study samples, dentin and enamel blocks of length and width of 5 mm and thickness of 2 mm were kept at a temperature of 40°C, a neutral pH of 7 in thymol solution (0.1%). All the samples were prepared from the maxillary incisors and from the middle third of the crown and were polished with carbide paper for grinding after being grounded. Then, the samples were placed in a diamond suspension.

Two of the prepared specimens were joined to form blocks for the study, which were then placed in a toothpaste slurry with tooth brushing on only one side. Following this, the blocks were divided into 10 groups to confirm randomization, and each group comprised a total of 8 study blocks. Except for the central part of the study, the blocks were covered with adhesive tape.

To evaluate the abrasive ability of the two kinds of toothpaste, the standard radioactive dentin abrasiveness (RDA) test was used where the automatic machine for tooth brushing was used for the 8 collected specimens using suspensions after mixing 20 grams of toothpaste in 45 ml of water (deionized) or by using the standard available calcium pyrophosphate abrasive. A total of 100-gram calcium pyrophosphate abrasive was used in the present study. The study protocol, as explained in Table 1, was based on the daily procedures, including the exposure of the study specimen to citric acid for 5 minutes to erode the sample, followed by allowed remineralization in artificial saliva for 1 hour. The next step was automatic brushing with a slurry of toothpaste (45 motions) containing fluoride for 15 seconds, followed by exposure to experimental mouthwash for 1 minute. Then, the 1-hour remineralization process was allowed in the artificial saliva. Four cycles of erosion and remineralization were allowed for the mentioned time repeatedly. The final steps were exposure to brushing with fluoridated toothpaste and mouthwash, and finally, remineralization in artificial saliva for a whole night.

**Table 1:** The study protocol.

	Treatment	Duration
**Step 1**	Erosion of specimen on account of citric acid	5 min
**Step 2**	Remineralization achieved with artificial saliva	60 min
**Step 3**	Exposure to fluoride toothpaste slurry was done by a brushing machine	15 sec (45 strokes)
**Step 4**	Exposure to treatment mouthwash	1 min
**Step 5**	Remineralization achieved with artificial saliva	60 min
**Step 6**	Erosion of specimen on account of citric acid	5 min
**Step 7**	Remineralization achieved with artificial saliva	60 min
**Step 8**	Erosion of specimen on account of citric acid	5 min
**Step 9**	Remineralization achieved with artificial saliva	60 min
**Step 10**	Erosion of specimen on account of citric acid	5 min
**Step 11**	Remineralization achieved with artificial saliva	60 min
**Step 12**	Erosion of specimen on account of citric acid	5 min
**Step 13**	Remineralization achieved with artificial saliva	60 min
**Step 14**	Exposure to fluoride toothpaste slurry with a brushing machine	15 sec (45 strokes)
**Step 15**	Exposure to mouthwash	1 min
**Step 16**	Remineralization achieved with artificial saliva	Overnight

After completion of the mentioned steps of daily procedures, an optical profilometer was used for a profilometry study to evaluate the surface loss after removing the adhesive tapes. Using a parallel surface as an experiment, the blocks were placed in an optical profilometer. Using two resolutions of 0.05 and 0.01 mm, the whole block area was exposed and scanned to assess the surface loss. The resolutions used were horizontally, allowing scanning on the x and y-axis. Before starting the scanning procedure, drying of the dentinal specimens was done for 10 minutes. This allowed the elimination of errors by organic compound shrinkage in the dentin. The relevant area was assessed and was removed from the reference area pair calculated by image analysis. The surface loss was evaluated as depth difference was calculated using a micrometer.

The data for enamel and dentin were evaluated separately to assess the surface loss due to the effects of tooth brushing, toothpaste, and mouth rinse. An ANOVA test was used to assess the collected data with overall significance kept at the level of 5% using the Sidak method for pair-wise comparison. The collected data were subjected to statistical evaluation to structure the results.

## RESULTS

The study results showed no interaction in the three methods evaluated, including mouthwash, kinds of toothpaste, and the surface loss/abrasion with a p-value of 0.4720 and 0.0520 for enamel and dentin, respectively (Table 2). A significant surface loss was seen with the abrasion caused by the toothbrush in enamel compared to dentin with a p-value<0.0001 in both enamel and dentin. The surface loss was not affected by any of the mouthwashes used in the study. More abrasion was seen with fluoridated toothpaste having sodium fluoride in comparison to the stannous fluoride. This difference between the two fluoridated toothpaste was statistically significant (p<0.0001).

**Table 2:** Mean abrasion value in dentin.

Test Article	Relative dentine abrasion
**Senquel F**	146.56±10.35
**Crest**	100.93±2.16

Enamel and dentin surface loss was significantly higher when surfaces were subjected to erosion along with abrasion compared to surfaces that were not exposed to abrasion (0.0001). On assessing the two-way interaction, the effect of only mouth rinse and toothpaste was seen on the surface loss with less surface loss with sodium fluo-ride (4.60 μm) compared to stannous fluoride (5.83 μm) (Table 3). This difference was statistically significant with p<0.0001).

**Table 3:** Results of the statistical analysis for surface loss of enamel; toothpaste and brushing effects.

Comparison	Result	Estimate	Std Err	P-value	Sig
**Toothpaste**	NaF>SnF	1.6430	0.2515	<.0001	*
**Brush/not**	No<Yes	-4.3048	0.2515	<.0001	*

* Statistically significant values.

Concerning dentin, it was noted that interaction was not seen between the assessed three factors, including mouth rinses, toothbrush/no tooth brushing, and fluoridated toothpaste with a p-value of 0.0520. The mean surface loss in dentin was significantly higher for the fluoridated toothpaste containing stannous fluoride compared to sodium fluoride (p<0.0001). For mouth rinses, the dentinal surface loss difference was statistically insignificant with a p-value of 0.9927. The surface loss for dentin was in the range of minimum values of -5.160 to -8.890 and a maximum of -0.223 to -4.765 with mean values in the range of 3.653 to -6.422, as shown in Table 4.

**Table 4:** Summary of the statistical results for dentin surface loss.

Toothpste	Rinse	Brush/not	No.	Mean	SD
	CHX	No	8	-3.809	1.318
		Yes	8	-4.379	2.375
	CPC	No	8	-3.955	1.734
		Yes	8	-5.824	0.993
**NaF**	D/I	No	8	-3.653	1.709
		Yes	8	-4.556	1.306
	EO	No	8	-3.407	1.332
		Yes	8	-6.422	1.451
	F	No	8	-4.033	1.567
		Yes	8	-6.022	1.561
**SnF2**	CHX	No	8	-4.467	1.225
		Yes	8	-7.905	2.112
	CPC	No	8	-4.902	0.955
		Yes	8	-6.424	1.905
	D/I	No	8	-5.828	1.046
		Yes	8	-6.682	1.500
	EO	No	8	-5.135	1.818
		Yes	8	-6.258	1.929
	F	No	8	-4.555	1.198
		Yes	8	-6.142	1.724

For enamel surfaces, it was noted that interaction was not seen between the assessed three factors, including mouth rinses, toothbrushing/no tooth brushing, and fluoridated toothpaste with a p-value of 0.4720. In contrast, the mean value of surface loss for enamel was higher for fluoridated toothpaste with sodium fluoride than the stannous fluoride, which was statistically significant (p<0.0001). A non-significant difference was seen in all mouthwashes used in the study with a p-value of 0.1946, as depicted in Table 5.

**Table 5:** Summary of the statistical results for enamel surface loss.

Toothpaste	Rinse	Brush/not	No.	Mean	SD
	CHX	No	8	-2.318	0.522
		Yes	8	-8.040	1.490
	CPC	No	8	-2.834	1.370
		Yes	8	-6.140	3.017
**NaF**	D/I	No	8	-3.437	1.145
		Yes	8	-7.416	1.246
	EO	No	8	-3.737	0.420
		Yes	8	-7.761	2.352
	F	No	8	-3.132	0.610
		Yes	8	-7.180	1.948
**SnF2**	CHX	No	8	-1.380	0.300
		Yes	8	-5.561	1.788
	CPC	No	8	-1.051	0.965
		Yes	8	-5.357	2.607
	D/I	No	8	-1.790	0.538
		Yes	8	-6.260	2.095
	EO	No	8	-0.952	0.362
		Yes	8	-4.898	2.183
	F	No	8	-1.624	0.732
		Yes	8	-6.692	1.803

The study results depicted that the surface loss of dentin was lower with fluoridated toothpaste containing sodium fluoride compared to stannous fluoride. In contrast, surface loss in enamel was significantly higher with fluori-dated toothpaste containing sodium fluoride compared to stannous fluoride. Additively, for both dentin and enamel, it was seen that surface loss was higher significantly in erosion in addition to abrasion compared to that without tooth brushing. Also, no statistically significant difference with the use of any mouth rinses was seen on the enamel or dentin surface loss.

## DISCUSSION

The present study followed a pre-decided protocol based on 5-day cycles of abrasion and erosion with periods of erosion in citric acid, followed by remineralization using artificial saliva, abrasion using toothpaste slurry, and exposure to mouth rinses.

The present study assessed the surface loss following the use of toothpaste and tooth brushing, and the statistically significant difference was seen in dental and enamel blocks with a p-value<0.0001. The comparison was carried out between the no brushing and tooth brushing groups. These findings were consistent with the findings of the studies by Magalhães *et al*. [12] and Rios *et al*. [13], where the authors reported similar findings. Regarding surface loss, more resistance was seen in dentin compared to enamel with sodium fluoride-containing toothpaste. The mechanism behind resistance provided by sodium fluoride can be owed to the formation of a resistant layer rich in fluoride, which acts as protection against acid exposure [14]. The present study results agree with the study of Dehghan *et al*. from 2017, where authors described enamel loss depth [15]. Enamel loss was significantly higher in groups where the toothpaste was used with brushing compared to water and brush. Besides, after exposure to artificial saliva for 60 minutes, the surface loss for enamel was decreased significantly using brush and water but not using the toothpaste. The study evaluated surface loss resulting from erosion after abrasion by brushing; the results showed that toothpaste caused surface abrasion on enamel surfaces exposed to citric acid.

Similar to the present study, a study by Kumar *et al*. showed that tooth brushing with water involved less surface loss compared to the use of toothpaste with a p-value<0.008 [16]. When using water with a toothbrush, greater abrasion was shown by a hard-bristled toothbrush, whereas a soft-bristled brush showed more abrasion when using the toothpaste. Also, a study conducted by Muntean *et al*. in 2019 showed that fluoride leads to mineral absorption in the enamel surface lacking minerals [17].

The present study also evaluated the effect of various mouth rinses on the protection to surface loss provided by toothpaste containing fluorides. The study results showed that all the tested mouth rinses used (essential oils, fluo-ride, CPC, distilled water, or chlorhexidine) showed no statistically significant difference in terms of evaluated fluoride for their capability in surface loss from erosion. The study specimens were immediately treated with mouth rinses after brushing with fluoridated toothpaste which led to the increased fluoride release from the study specimen, which in turn reduced the efficiency.

No statistically significant difference was seen in the present study when distilled water was compared to the mouth rinses containing sodium fluoride with p-values of 0.196 and 0.9927 for enamel and dentin, respectively. This might be attributed to the fact that, in low concentration, fluoride provides no resistance against surface loss by erosion, and study blocks probably lost their ability to absorb fluo-ride after exposure to fluoridated toothpaste. The present study also showed that chlorhexidine mouth rinse did not affect the action of fluoridated toothpaste against surface loss, which can be due to loss of fluoride following rinsing that further reduced fluoride retention.

The study of Duckworth *et al*. from 2009 also showed similar results to the present study, stating that rinsing with a mouth rinse containing no fluoride after brushing with a fluoridated toothpaste can markedly decrease protection against caries and erosion provided by fluoridated toothpaste alone [18].

The present study showed that no significant difference was seen in any mouth rinse with CPC, essential oils, or chlorhexidine in terms of their effect on a fluoridated toothpaste in preventing tooth surface loss. These findings were consistent with the study conducted by Latimer *et al*. in 2015, where the authors found no significant difference in water and essential oil rinses on erosion and no effect of sodium fluoride on the ability to prevent the surface loss, suggesting the usage of a combination of sodium fluoride with CPC for protection against surface loss and antimicrobial action [19].

This study had a few limitations, including that the study was conducted in an in-vitro artificial environment, and the natural oral environment, including soft tissues and oral mucosa, was not considered. Also, the study showed no effect of various mouth rinses, which might differ in the oral environment owing to its retention on oral surfaces as tongue and mucosa. Retention can also be increased in a larger oral cavity compared to study specimens.

Hence, further investigation is needed. In the present study, constant and fixed time was used for rinsing following brushing, which is not feasible in vivo. Future research should focus more on the varying time between brushing and rinsing.

## CONCLUSION

The present study utilized a standard cycle model on abrasion or erosion to evaluate the effect of different mouth rinses on increasing the ability of toothpaste containing strontium fluoride and sodium fluoride to decrease tooth surface loss.

With its mentioned limitations, the study concluded that various mouth rinses with different compositions and main ingredients did not affect the protective actions of fluoridated toothpaste on tooth surface loss. Concerning dental erosion, better protection against surface loss was seen with fluoridated toothpaste containing strontium fluoride compared to sodium fluoride. For only dentin erosion, the tested NaF dentifrice offered greater protection against surface loss compared to that containing strontium fluoride. Abrasion caused by tooth brushing also led to the increased surface loss in subjects with previously eroded dentin and enamel.
